# The effects of vitamin-rich carbohydrate pretreatment on the surgical stress response and S-100β after splenectomy in elderly rats

**DOI:** 10.1186/s12871-019-0748-0

**Published:** 2019-05-15

**Authors:** Youbo Zuo, Lei Zhao, Mei Zeng, Qiuyan Yang, Xueli Chen, Tiande Yang

**Affiliations:** 1 Department of Anesthesiology, Xinqiao Hospital, Army Medical University (Third Military Medical University), Chongqing, 400037 China; 2 Department of Anesthesiology, Affiliated Hospital of North Sichuan Medical College, Nanchong, 637000 China; 3 Department of Genetics, School of Basic Medical Science, North Sichuan Medical College, Nanchong, 637007 China; 4 Department of Emergency, Affiliated Hospital of North Sichuan Medical College, Nanchong, 637000 China; 5 Department of Anesthesiology, North Sichuan Medical College, Nanchong, 637007 China

**Keywords:** Carbohydrate, Insulin resistance, Surgical stress response, Inflammatory mediators, S-100β

## Abstract

**Background:**

Preoperative oral carbohydrates has been suggested to attenuate insulin resistance and decrease postoperative complications. In this study, a vitamin-rich carbohydrate beverage was administered before surgery in an animal model to investigate its effects on the surgical stress response and S-100β levels.

**Methods:**

Thirty aged male Sprague-Dawley rats were randomly assigned to three groups: control group (*n* = 6), fasting group (*n* = 12), and carbohydrate-treated group (CHO group, *n* = 12). Rats in the control group were not given any treatment. Rats in the fasting group received splenectomy after 12 h of fasting. In the CHO group, rats were given 5 ml of vitamin-rich carbohydrate by gavage 2 h before surgery. Fasting plasma glucose, insulin, insulin resistance (HOMA-IR index, IRI), the S-100β protein level, and the inflammatory mediators IL-1β, IL-6 and TNF-α were assessed after surgery (postoperative day (POD) 1 and 3).

**Results:**

Postoperative insulin resistance was significantly greater in the fasting group than in the control and CHO group. The median plasma S-100β level was significantly higher in the fasting group than in the control and CHO groups on POD 1. The median plasma IL-1β level was significantly lower in the CHO group than in the fasting group on POD 1; however, no other differences in the concentrations of immunological biomarkers of stress were found between the fasting group and the CHO group.

**Conclusions:**

Vitamin-rich carbohydrate pretreatment attenuated the metabolic aspect of the surgical stress response and decreased the level of plasma S-100β, which may decrease the risk of postoperative complications in elderly rats.

## Background

The surgical stress response disrupts metabolic homoeostasis and induces insulin resistance. The previously fasting guideline recommended fasting on the night before surgery, which intensifies postoperative insulin resistance (PIR) [1–3]. PIR is a pivotal feature of postoperative metabolic response, and can reduce insulin-stimulated glucose uptake in skeletal muscle and adipose tissue, and increase glucose release, which may lead to hyperglycemia [4]. The adverse effects of PIR and hyperglycemia may increase postoperative complications, including infection and organ dysfunction, eventually extending the hospital stay and increasing morbidity [5]. Tumor necrosis factor-α (TNF-α) and interleukin-6 (IL-6), as important pro-inflammatory cytokines, correlate well with the extent of tissue trauma [6] and the magnitude of PIR [7]. Both insulin resistance and systemic inflammatory cytokines are related to the surgical stress response and postoperative complications [5, 8, 9].

As one part of the Enhanced Recovery After Surgery (ERAS) protocol, the intake of clear fluids up to 2 h before induction of anesthesia for elective surgery has been recently recommended [10]. A carbohydrate-rich beverage, one type of clear fluid, is the most efficient and natural way to provide certain quantities of carbohydrates (CHO) without any threat of aspiration [11]. Preoperative carbohydrate treatment has been reported to reduce insulin resistance [12, 13], decrease circulating IL-6 concentrations [14], improve patient well-being [15], maintain postoperative whole-body protein balance and muscle strength [16], and result in a shorter hospital stay. However, its effects on the surgical stress response and postoperative complications are still under debate [17]. Additionally, a well-established animal model to investigate this mechanism is lacking.

The present study aims to describe the surgical stress response in elderly rats undergoing splenectomy, and to investigate whether vitamin-rich carbohydrate pretreatment modifies the surgical stress response and the plasma level of S-100β.

## Methods

### Animals

Thirty male Sprague-Dawley (SD) rats (weight, 500–650 g; age, 18–20 months) were supplied by the Laboratory Animal Center of North Sichuan Medical College. All animals were maintained in a temperature, and humidity-controlled room (21 ± 2 °C and 55 ± 5%, respectively), with 12 h light/12 h dark cycles, and free access to food and water. The experiments were approved by the Animal Care Committee of North Sichuan Medical College, and the experimental procedures were carried out in accordance with the *Guide for the Care and Use of Laboratory Animals* published by the National Institutes of Health (NIH Publication No. 85–23, revised 1996).

### Main experimental materials and reagents

Operative devices designed for small animals were used in this study. The vitamin-rich CHO beverage (14.2% carbohydrates, Outfast, YICHANG HUMANWELL PHARMACEUTICAL CO., LTD. China) used in this study contained water, maltodextrin, crystalline fructose, glucose, food additives (sodium citrate, citric acid monopotassium phosphate, potassium sorbate, and L-malic acid), taurine, zinc gluconate, vitamin B_1_, vitamin B_6_, vitamin B_12,_ and flavoring.

The Rat/Mouse insulin enzyme-linked immunosorbent assay (ELISA) kit (sensitivity, 2–600 mIU/L), and Rat Soluble protein-100 ELISA kit (sensitivity, 20–6000 ng/L) were purchased from Nanjing Jiancheng Bioengineering Institute. The Rat/Mouse TNF-α, IL-1β, and IL-6 ELISA kits (sensitivity, < 15 pg/ml) were purchased from Beijing 4A Biotech Co., Ltd. The protocols were conducted according to the instructions of the corresponding kits.

### Experimental protocol and surgical procedures

All rats were randomly assigned to three groups: control group (*n* = 6), fasting group (*n* = 12), and carbohydrate-treated group (CHO group, *n* = 12). The rats in the control group were not given any treatment. The rats in the fasting group were fasted for 12 h, and then splenectomy was performed. In the CHO group, rats were fasted for 10 h and then were given 5 ml of vitamin-rich carbohydrate by gavage 2 h before splenectomy.

For the splenectomy, rats were anesthetized with an intraperitoneal injection of pentobarbital sodium (35 mg/kg). After successful anesthesia induction, animals were fixed on the operating table. The entire process was performed under sterile conditions with the abdominal skin sterilized by 5% iodophor. The spleen was exposed through a 2 cm incision in the left upper abdominal quadrant; and was then removed from the abdomen. The blood vessels of the spleen were ligated using 7–0 silk sutures, and the spleen was removed by transecting the blood vessels near the spleen. The wound was infiltrated with 0.25% bupivacaine and closed using sterile sutures.

The surgical process lasted approximately 30 min. The animals were housed individually in cages with free access to standard rat chow and tap water after surgery.

### Detection methods

Rats were sacrificed with sodium pentobarbital via an intraperitoneal injection (60 mg/kg) on postoperative days 1 and 3. Blood was sampled from an incision in the femoral artery, which was then quickly dissected. The blood glucose level was measured immediately using the glucose oxidase method (Accutrend Alpha, Roche, Switzerland). The remaining plasma was stored at − 70 °C until it was used for other measurements. Changes in the plasma levels of insulin, TNF-α, IL-1β, IL-6 and S-100β were detected by ELISA according to the manufacturer’s instructions, after all samples were collected. For assessment of insulin resistance, the homoeostasis model assessment (HOMA-IR) was used, as in humans, according to the following formula: HOMA-IR = (blood glucose (mmol/l) × blood insulin (μunits/ml))/22.5. Similarly, the insulin sensitivity index (ISI) was determined according to the following formula: ISI = 1/(lg(blood glucose (mmol/l)) + lg(blood insulin (μunits/ml))) [13].

### Statistical analysis

All data were analyzed by SPSS 17.0 statistical software. Quantitative data are expressed as medians (range). Comparisons between the fasting group and the CHO group at corresponding times, and comparisons between the control group and other groups, were performed with the Wilcoxon and Mann-Whitney *U* test. A value of *P* < 0.05 was considered statistically significant.

## Results

### Vitamin-rich carbohydrate beverage attenuated insulin resistance

**Fig. 1:**
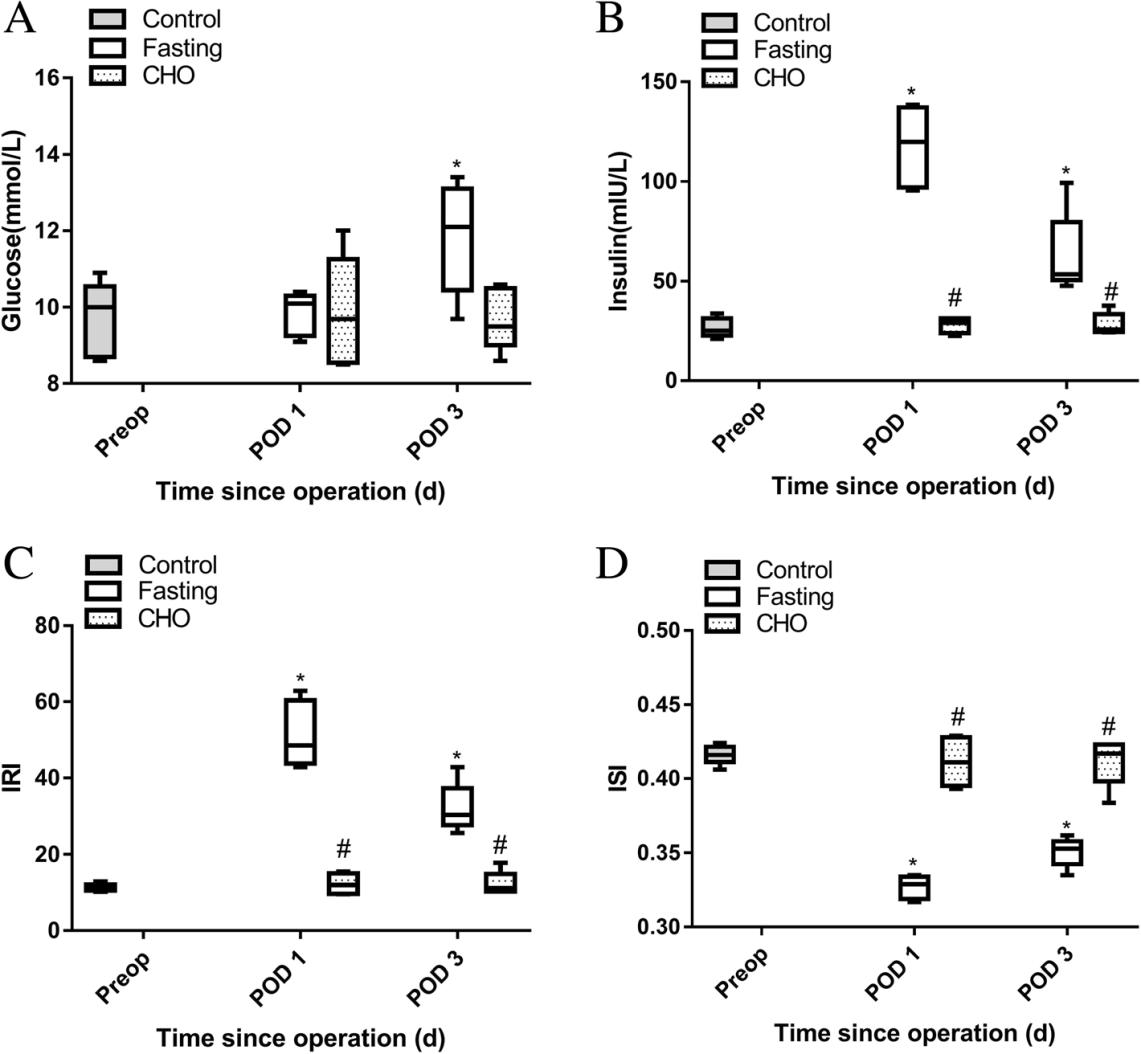
Postoperative insulin resistance in elderly rats undergoing splenectomy: **a** plasma glucose; **b** plasma insulin; **c** the homoeostasis model assessment of insulin resistance (HOMA-IR); **d** insulin sensitivity index (ISI). Values are shown as medians, interquartile intervals and ranges. ^*^P < 0.05 vs. the control group; ^*#*^ *P* < 0.05 vs. the corresponding day in the fasting group, *n* = 6

The median plasma glucose levels had significantly increased on POD 3 in the fasting group (*P* < 0.05) (Fig. 1a). The median plasma insulin levels and median levels of HOMA-IR had increased significantly on POD 1 and POD 3 in the fasting group, and were significantly higher than those in the CHO group (*P* < 0.05) (Fig. 1b, c). The median ISI values of rats in the CHO group were significantly higher on POD 1 and POD 3 than those in the fasting group (*P <* 0.05) (Fig. 1d). These results indicated that the vitamin-rich carbohydrate beverage attenuated postoperative insulin resistance and preserved postoperative insulin sensitivity.

### Postoperative inflammatory response in elderly rats undergoing splenectomy

**Fig. 2:**
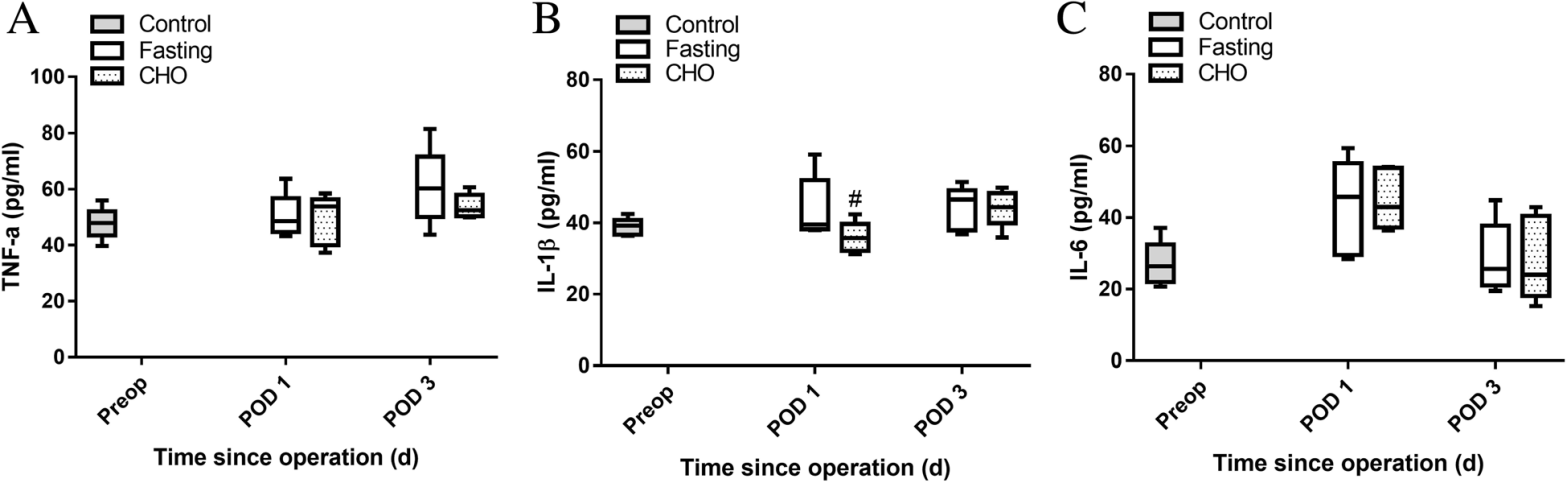
Postoperative inflammatory response in elderly rats undergoing splenectomy: **a** plasma TNF-a; **b** plasma IL-1β; **c** plasma IL-6. Values are shown as medians, interquartile intervals and ranges. ^*#*^ *P* < 0.05 vs. the corresponding day in the fasting group, *n* = 6

The median plasma IL-1β level on POD 1 in the CHO group was significantly lower than that in the fasting group (*P* < 0.05) (Fig. 2b). However, no differences in median plasma TNF-α and IL-6 levels were observed between the fasting group and the CHO group. These results indicated that the effect of the vitamin-rich carbohydrate beverage on inflammatory response is inexact.

### Vitamin-rich carbohydrate reduced the plasma concentration of S-100β

**Fig. 3:**
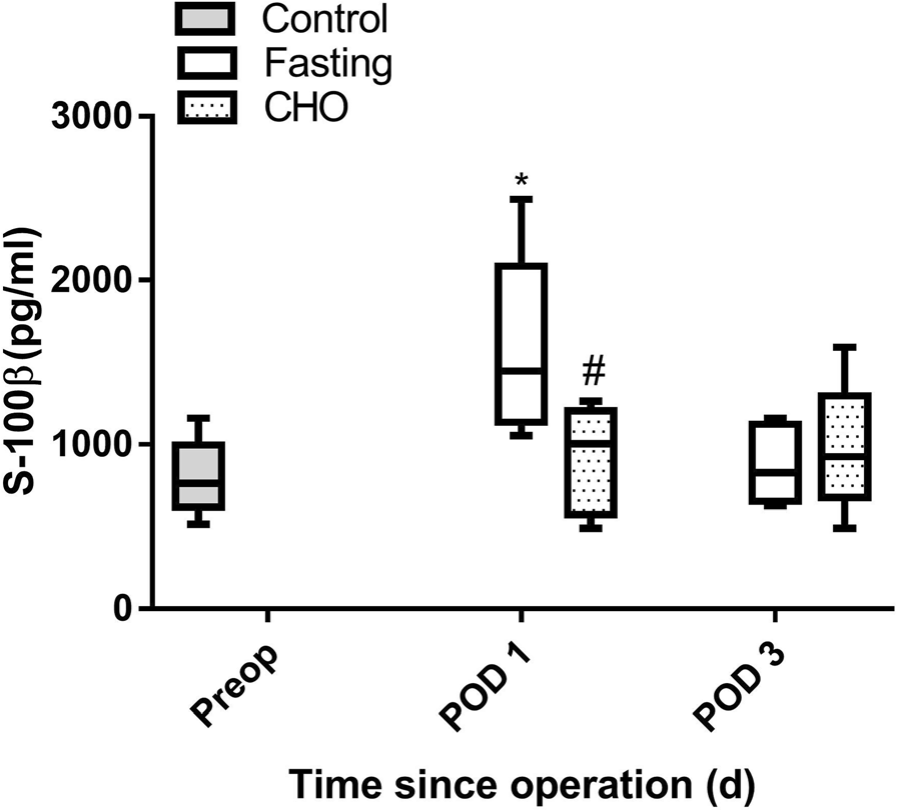
Plasma S-100β expression in elderly rats undergoing splenectomy. Values are shown as medians, interquartile intervals and ranges. ^*^P < 0.05 vs. the control group; ^*#*^ *P* < 0.05 vs. the corresponding day in the fasting group, *n* = 6

The median plasma S-100β level in the fasting group was increased on POD 1. However, the S-100β level in the CHO group was not increased on POD 1, and was significantly lower than that in the fasting group on POD 1 (*P* < 0.05) (Fig. 3). The results indicated that the vitamin-rich carbohydrate beverage inhibited S-100β production.

## Discussion

Modulation of surgical stress responses is the core aspect of the ERAS protocol. Surgical stress responses involve the metabolic, neuroendocrine and immunological systems, and include postoperative insulin resistance (PIR) and inflammatory reaction. Excessive inflammatory responses and PIR may lead to immunodepression and increase the risk of postoperative complications, such as infection and organ damage. This study showed that vitamin-rich carbohydrate pretreatment attenuated PIR and decreased the plasma levels of S-100β in rats undergoing splenectomy.

The results of our study suggested that pretreatment with 5 ml of a vitamin-rich carbohydrate beverage (approximately 1 ml/100 g) effectively improved PIR and insulin sensitivity in rats undergoing splenectomy. Similar patterns were also observed in other studies [18, 19]. It is well known that surgery induces insulin resistance, which impairs the effects of insulin on protein and fatty metabolism. PIR, which is a marker of surgical stress [5], plays a pivotal role in the pathogenesis of postoperative complications and organ impairment, affecting postoperative recovery [20–22]. IR usually presents as high blood glucose and insulin levels. Furthermore, we found that the insulin level was markedly increased after surgery in the fasting group, especially on POD 1, and the level was significantly higher than that in the CHO group. The concentration of plasma glucose was not significantly increased in the fasting group on POD 1, which may be due to the high levels of plasma insulin. However, when the insulin level declined on POD 3, the glucose level in the fasting group, increased to a level higher than that in the control group. However, no difference was found in these parameters between the control group and the CHO group. The results indicated that pretreatment with a vitamin-rich carbohydrate beverage effectively attenuated PIR in rats undergoing splenectomy.

Viganò et al. found that preoperative carbohydrate supplementation could decrease IL-6 levels and attenuate the postoperative metabolic stress response of patients undergoing elective abdominal surgery [14]. Gjessing et al. also demonstrated that preoperative oral carbohydrate administration reduced muscle inflammatory responses in a pig model of major abdominal surgery [23]. Postoperative TNF-α, IL-1β and IL-6 are useful markers and are the most frequently studied cytokines of the surgical stress response. The concentrations of inflammatory mediators are direct indicators of perioperative stress [24]. An excessive inflammatory response to surgery has been related to infectious complications and tumor metastasis [25, 26], suggesting that control of inflammation may improve outcomes and reduce complications [27, 28]. However, our study failed to determine the exact effect of the vitamin-rich carbohydrate beverage on postoperative inflammatory responses, which may be related to the low number of rats in each group.

The brain is the most sensitive organ to energy metabolism; therefore, we measured plasma S-100β levels. S-100β is a commonly used nonspecific marker for brain injury that is useful in evaluating the severity of brain injury [29]. In the present study, we found that vitamin-rich carbohydrate pretreatment decreased the elevated plasma levels of S-100β protein that were exhibited by rats in the fasting group on POD 1, demonstrating that the vitamin-rich carbohydrate beverage may have a neuroprotective effect. S-100β protein is predominantly expressed and secreted by astroglial cells in the central nervous system; therefore, its noticeable presence in blood serum may reflect brain injury. The serum levels of S-100β increase after various types of trauma, such as head injury and major surgery [30, 31]. The changes in BBB permeability after surgical trauma have also been found in aged POCD rats [32]. Suppression of plasma S-100β levels may be related to a reduction in cerebral complications.

Our study found that administration of a vitamin-rich carbohydrate beverage (5 ml by gavage) could effectively attenuate postoperative insulin resistance, improve insulin sensitivity, and decrease the production of IL-1β and S-100β in the plasma of elderly rats after splenectomy. However, the effects of the vitamins and other components in the carbohydrate beverage require further study.

## Conclusion

In summary, pretreatment with a vitamin-rich carbohydrate beverage attenuates surgical stress responses in elderly rats, as indicated by PIR, and inhibits the release of plasma S-100β, which may result in improved postoperative outcomes and fewer complications.

## Abbreviations

CHO: carbohydrates
ELISA: enzyme-linked immunosorbent assay
IL-1β: interleukin 1-beta
IL-6: interleukin-6
S-100β: S-100 calcium-binding protein B
TNF-α: tumor necrosis factor-α
